# Development and Validation of the Intimate Partner Violence Nursing Competency Scale (IPVNCS): A Psychometric Tool to Strengthen Clinical Detection and Intervention

**DOI:** 10.3390/jcm15031001

**Published:** 2026-01-26

**Authors:** David Casero-Benavente, Natalia Mudarra-García, Guillermo Charneco-Salguero, Leonor Cortes García-Rodríguez, Francisco Javier García-Sánchez, José Miguel Cárdenas-Rebollo

**Affiliations:** 1 Department of Nursing, School of Medicine, Universidad San Pablo-CEU, CEU Universities, Urbanización Montepríncipe, 28660 Boadilla del Monte, Spain; david.caserobenavente@ceu.es (D.C.-B.); natalia.mudarragarcia@ceu.es (N.M.-G.); guillermo.charnecosalguero@ceu.es (G.C.-S.); leonor.garciarodriguez@ceu.es (L.C.G.-R.); 2 Instituto Ramón y Cajal de Investigación Sanitaria (IRYCIS), 28034 Madrid, Spain; 3 Group for Research in Community Care and Social Determinants of Health, University of Alcalá, 28805 Madrid, Spain; 4 Emergency Room Service, Surgical Prehabilitation Unit, Hospital Universitario Infanta Cristina, Instituto de Investigación Sanitaria Hospital Puerta de Hierro Segovia Arana (IDIPHISA), 28981 Madrid, Spain; 5 Medical Department, Faculty of Medicine, University Complutense of Madrid, 28040 Madrid, Spain; 6 Department of Mathematics and Data Science, School of Economics and Business Sciences, Universidad San Pablo-CEU, CEU Universities, Calle Julián Romea 22, 28003 Madrid, Spain; cardenas@ceu.es

**Keywords:** intimate partner violence, nursing, psychometric scale, nursing process

## Abstract

**Background**: Intimate partner violence (IPV) represents a major public health problem in Europe, with significant physical, psychological, and social consequences. Nurses are often the first professionals capable of detecting early signs of IPV, yet they lack validated instruments to assess their clinical competency in detection, evaluation, documentation, and intervention. This study aimed to develop and validate the Intimate Partner Violence Nursing Competency Scale (IPVNCS), aligned with the Nursing Intervention Classification (NIC 6403). **Methods**: A cross-sectional psychometric study was conducted among registered nurses in the Community of Madrid. A 30-item Likert-type self-administered instrument (1–5 scale) was developed based on NANDA, NIC 6403, and NOC frameworks. A total of 202 nurses participated. Reliability was assessed through Cronbach’s alpha. Construct validity was examined using exploratory factor analysis (EFA) with Promax rotation and confirmatory factor analysis (CFA) using AMOS 26. Ethical approval was obtained (CEU San Pablo, code 843/24/104). **Results**: After item refinement, 26 items remained across four dimensions: (1) Intervention and Referral, (2) Detection and Assessment, (3) Documentation and Recording-keeping, (4) Psychosocial Support. The instrument showed excellent reliability (α = 0.97). KMO was 0.947 and Bartlett’s test was significant (*p* < 0.001). CFA demonstrated satisfactory fit: $\chi^{2}$/df = 2.066, RMSEA = 0.073, CFI = 0.92, TLI = 0.91, NFI = 0.86. The final model adequately represented the latent structure. After debugging, its psychometric properties were significantly improved. Four redundant items were eliminated, achieving internal consistency (α = 0.97), a KMO value of 0.947 and a significant Bartlett’s test of sphericity. It showed a better fit, according to $\chi^{2}$/df = (2.066); Parsimony = (720.736); RMR (0.0529; RMSEA (0.073); NFI (0.860); TLI (0.910) and CFI (0.920). The final model provides an adequate representation of the latent structure of the data. This study provides initial evidence of construct validity and internal consistency reliability of the IPVNCS. **Conclusions**: The IPVNCS is a valid and reliable tool to assess nursing competencies for clinical management of IPV. It supports structured evaluation across four core nursing domains, enabling improved educational planning, clinical decision-making, and quality of care for victims. The scale fills a gap in clinical nursing assessment tools and can support protocol development in emergency, primary care, and hospital settings.

## 1. Introduction

Intimate partner violence (IPV) is a critical public health issue in Europe, affecting millions of individuals each year. It includes physical, psychological, sexual, and economic abuse, with women being disproportionately affected. IPV is associated with chronic physical conditions, mental health disorders, and increased healthcare utilization. Improved detection within clinical settings is essential to mitigate these consequences.

Nurses are uniquely positioned for early identification due to their continuous proximity to patients in emergency departments, primary care, and inpatient settings. However, several barriers—including insufficient training, lack of protocols, and limited self-confidence—reduce their ability to identify and manage IPV effectively. Current literature indicates a lack of validated nursing-focused tools to assess clinical competencies regarding IPV detection, assessment, documentation, and intervention.

This study addresses this gap by developing and validating the Intimate Partner Violence Nursing Competency Scale (IPVNCS), which is grounded in NANDA diagnoses [1], NOC outcomes [2], and NIC 6403 [3] (“Abuse Protection Support: Partner”) (Table A1). The objective is to provide a psychometrically robust instrument to evaluate nurses’ perceived competency in managing IPV.

Despite advances in gender equality and efforts to eradicate the problem, it persists and manifests itself in various forms, ranging from physical to psychological and sexual violence. Women are the primary victims, although men can also suffer from it. The consequences of intimate partner violence are devastating for the physical and mental health of the victims, as well as for their social and economic well-being. It is essential to address this problem from a multidisciplinary perspective by implementing effective public policies and strengthening support systems for victims [4]. Nurses, because of their direct contact with patients, are therefore key to the early detection of intimate partner violence. However, they face challenges such as a lack of specific training and resources. Therefore, it is essential to have assessment tools that allow them to effectively identify situations of violence, make a nursing diagnosis, and provide comprehensive care to victims. Intimate partner violence (IPV) presents various factors, such as a history of child abuse [5] or low self-esteem, that increase vulnerability to this issue. Nurses must be aware of the cycle of violence and the risk factors in order to provide appropriate and personalized care.

The devastating consequences of intimate partner violence on physical and mental health make an effective response essential. Nurses, being in direct contact with the victims, are key to detecting and addressing this problem. The implementation of specific assessment instruments, the continuous training of active professionals, and university training at the degree level [6] are essential to improving the response to this public health problem. Therefore, this study aims to develop a scale with high validity and reliability. The IPVNCS does not assume that nurses are competent; rather, it aims to identify whether and in which specific domains competency gaps may exist in IPV detection and management, in order to guide targeted training and organizational interventions.

## 2. Materials and Methods

### 2.1. Study Design

A cross-sectional psychometric validation study was conducted in 2024 in the Community of Madrid. Ethical approval was granted by CEU San Pablo University (843/24/104). During the preparation of this manuscript, Perplexity Pro (Perplexity AI, Inc., 115 Sansome St, Suite 900, San Francisco, CA 94104, CA, USA) was used to improve the English language and readability of the text. The authors reviewed and edited the output and take full responsibility for the final content of the publication.

### 2.2. Participants and Sample

Participants were recruited through professional nursing organizations, healthcare centers, and institutional communication channels. Participation was entirely voluntary, and no incentives were offered.

A total sample of 202 participants was obtained, meeting the recommended 5–10 subjects per item for factor analysis.

#### 2.2.1. Inclusion Criteria

Active clinical practice, an officially recognized nursing degree in Spain, and professional registration.

#### 2.2.2. Exclusion Criteria

Exclusion criteria included retired or inactive nurses, non-accredited degrees, and nursing students.

### 2.3. Measuring Instrument

A 30-item Likert-type scale (1 = not competent; 5 = highly competent) was created based on:

- NANDA: Dysfunctional Family Processes (00063).
- NIC 6403: Abuse Protection Support (partner).
- NOC 2603: Family Integrity.
- Clinical guidelines for the detection and management of IPV.

The scale covered detection, assessment, psychosocial support, documentation, and referral.

### 2.4. Description of the IPVNCS

The Intimate Partner Violence Nursing Competency Scale (IPVNCS) is a self-administered instrument designed to assess perceived nursing competencies in the clinical management of intimate partner violence (IPV). Importantly, the IPVNCS does not assume that nurses are competent in IPV detection and intervention; rather, it aims to identify whether and in which specific domains competency gaps may exist in order to guide targeted educational, organizational, and clinical improvement strategies.

The initial version of the IPVNCS consisted of 30 items developed based on the NANDA–NOC–NIC framework, with particular alignment to NIC 6403 (“Abuse Protection Support: Partner”). Items were formulated to reflect routine nursing activities related to IPV across different clinical settings.

Each item is rated on a five-point Likert scale, ranging from 1 (“Not competent at all”) to 5 (“Highly competent”), indicating the respondent’s perceived level of competency in performing the specified activity. Completion of all items was mandatory within the electronic questionnaire, which prevented missing data.

Following exploratory and confirmatory factor analyzes, four core competency dimensions were identified:

1. Detection and Assessment of Abuse: items addressing the ability to recognize physical, psychological, sexual, and social indicators of IPV and to assess risk factors. Example item: “I am able to identify clinical and behavioral indicators suggestive of intimate partner violence.”
2. Documentation and Record-keeping: items related to accurate, standardized, and legally appropriate documentation of suspected or confirmed IPV cases. Example item: “I am competent in documenting suspected IPV cases using standardized clinical records.”
3. Psychosocial Support: items reflecting the capacity to provide emotional support, encourage disclosure, and facilitate coping strategies for victims. Example item: “I am able to provide emotional support to patients who disclose intimate partner violence.”
4. Intervention and Referral: items addressing safety planning, referral to specialized services, and coordination with multidisciplinary teams. Example item: “I am able to initiate appropriate referrals to social, legal, or specialized healthcare services for patients experiencing IPV.”

The final validated version of the IPVNCS comprised 26 items, with four items removed due to redundancy and low contribution to model fit. The complete English version of the scale is provided as Supplementary Table S1.

### 2.5. Data Collection

The questionnaire was distributed electronically using Microsoft Forms^TM^, and nurses could complete it at their convenience. Completion of all items was required by the platform in order to submit the questionnaire, which prevented missing data. No identifying information was collected. Data were collected between June and December 2024.

### 2.6. Statistical Analysis

Analyzes were conducted using SPSS 29^TM^ and AMOS 26^TM^. Reliability: Cronbach’s alpha. Construct validity:

- EFA with Promax rotation: The number of factors to be retained was determined based on a combination of eigenvalues greater than one, inspection of the scree plot, and theoretical interpretability of the factor structure. Missing data were not present, as completion of all items was mandatory within the electronic questionnaire.
- Kaiser–Meyer–Olkin (KMO) and Bartlett’s test.
- CFA using maximum likelihood estimation.
- Internal consistency was assessed using Cronbach’s alpha for both the total scale and each identified subscale.

Fit indices included $\chi^{2}$/df, RMSEA, RMR, NFI, TLI, and CFI.

## 3. Results

### 3.1. Descriptive Data

The profile of the sample reveals a clear female predominance (81.7%), with a majority concentration in the 20–30 and 31–40 age groups. In terms of education, the high percentage of participants with Bachelor’s degrees and diplomas stands out, while doctorates represent a minority. Work experience is relatively evenly distributed among the different age groups, although there is a slight tendency towards those with more than 16 years of experience. With regard to the main occupation, primary care and hospital care account for most of the sample, while other areas such as teaching and social care are less represented (Table 1).

### 3.2. Exploratory Factor Analysis

An exploratory factor analysis with Promax rotation was conducted to identify the underlying structure of the 30 items in a sample of 202 participants. The analysis revealed a four-factor solution (Table 2). Promax rotation was selected to allow for correlations between factors, which is consistent with the multidimensional nature of the constructs assessed (Table 3). The resulting four factors exhibited high and consistent factor loadings. The IPVNCS demonstrated high internal consistency for the total scale (Cronbach’s α = 0.970). Subscale reliability was also high, with Cronbach’s α values ranging from 0.888 to 0.919 across the four dimensions (Table 4). The adequacy of the data for factor analysis was confirmed by the Kaiser–Meyer–Olkin index (KMO = 0.947) and Bartlett’s test of sphericity (*p* < 0.001) (Table 5 and Table 6). These results support the proposed factor structure of four identified dimensions: Dimension 1: Intervention and referral (items 11; 19; 26; 27; 28; 29); Dimension 2: Detection and assessment of abuse (items 1; 2; 3; 4; 16; 17); Dimension 3: Documentation and record-keeping (items 6; 7; 8; 9; 10; 13; 14; 15) and Dimension 4: Psychosocial support (items 5; 12; 20; 21; 22; 23).

### 3.3. Confirmatory Factor Analysis

**Figure 1:**
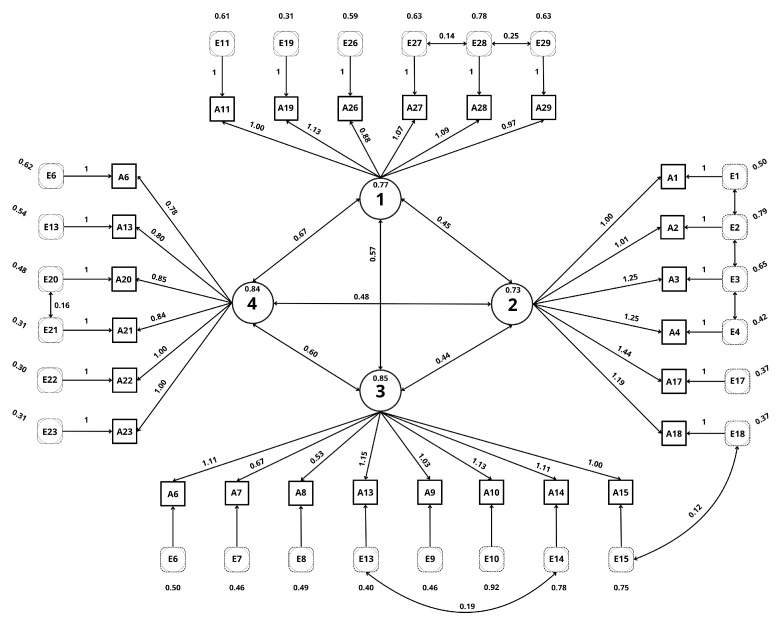
AFC diagram; Confirmatory factor analysis diagram representing the four-factor structure of the Intimate Partner Violence Nursing Competency Scale (IPVNCS).

A confirmatory factor analysis was conducted to identify the underlying structure of the variables. Two models were compared: an initial model and a final model, obtained after the elimination of items with low factor loadings and high communality. The final model showed a better fit according to $\chi^{2}$/df = 2.066; Parsimony = 720.736; RMR = 0.52; RMSEA (0.073); NFI (0.86); TLI (0.91); and CFI (0.92). These results suggest that the final model provides an adequate representation of the latent structure of the data (Table 7). For this purpose, items 18, 24, 25 and 30 were removed for redundancy (Figure 1).

These removed items correspond to the initial 30-item version and are not included in the final 26-item scale presented in Supplementary Table S1.

## 4. Discussion

The instrument obtained from the present study demonstrates that the refinement of the initially developed scale has significantly improved its psychometric properties. This study represents an initial validation of the IPVNCS, providing evidence of construct validity through exploratory and confirmatory factor analyzes, as well as internal consistency reliability. By eliminating 4 redundant items, a superior internal consistency (α = 0.97) and a clearer factor structure have been achieved, supported by a KMO value of 0.947 and a significant Bartlett’s test of sphericity. This optimization is in line with existing literature, which emphasizes the importance of reducing redundancy to improve the accuracy and discriminant validity of scales [7]. The findings of this study may have important implications for assessing the nursing profession’s knowledge of intimate partner violence intervention. Given the scarce existence of tools for measuring professional practice, with the focus of the obtained scales being victim-oriented [8], the professional’s attitude is extremely important for managing social and health resources [9]. The refined scale allows for a more accurate assessment of the proposed dimensions, facilitating its application in clinical settings as an element of management and training of human resources, as well as part of research and undergraduate and postgraduate education. As we can see in the following study [10] on gender-based violence among healthcare students and professionals currently practicing [10], the lack of perception of this issue and the lack of knowledge about it are once again highlighted. The refined version of the scale provides an efficient tool for assessing nursing competencies related to intimate partner violence and may contribute to improving both clinical practice and care management.

For all these reasons, it is especially beneficial in clinical, research, and teaching contexts, as well as in assessing the quality of care for patients who are victims of intimate partner violence. A brief and specific scale facilitates the application and interpretation of results without sacrificing precision, allowing for the evaluation of the strengths of health professionals within the nursing field to train with precision. As we saw in the study [11], the results indicated that training to detect and how to act was deficient, along with institutional barriers to case identification: ‘lack of time in the consultation (27.2%), absence of detection protocols for case management (22.8%), saturation of health services (22.8%). Among the personal barriers to GBV were: the disinterest of health personnel in identifying cases of GBV (29.4%) [11]. With this scale, it is possible to measure the dimension affected within the nursing process (NP) and to act more specifically to solve the barriers.

To our knowledge, most existing instruments focus primarily on victims, whereas the IPVNCS addresses nursing competencies related to the comprehensive management of intimate partner violence [12] as can be seen in ‘Training should not be restricted or focused exclusively on violence against women; rather, it should be placed in the broader context of the study of gender inequalities in health and the biases arising from the lack of this perspective in both clinical practice and epidemiological research’.

Finally, these data suggest that the scale is a valid and reliable tool for assessing nursing competencies in the field of detection, intervention, and support for victims of intimate partner violence. Robust psychometric properties were obtained, allowing for an accurate and structured assessment of the skills of nurses, with positive implications for improving the care and management of intimate partner violence cases in the healthcare setting. The refinement of these items allowed for the optimization of the accuracy and relevance of the factors measured by the scale, reducing the overlap of results.

Importantly, the IPVNCS is intended as a formative and supportive tool rather than a punitive or evaluative instrument. Its primary purpose is to guide professional development and identify training needs, particularly among early-career nurses, and to support institutional strategies aimed at improving care for individuals experiencing intimate partner violence.

## 5. Limitations

This study has several limitations that should be considered when interpreting the results. First, the validation was conducted using a single convenience sample of nurses from the Community of Madrid, which may limit the generalizability of the findings to other geographic, cultural, or healthcare contexts. Replication studies in different regions and healthcare systems are therefore warranted.

Second, both the exploratory and confirmatory factor analyzes were performed using the same sample. While this approach is acceptable for initial scale development and refinement—particularly to assess model fit after item reduction—it may inflate model fit indices. External validation using an independent sample would provide stronger evidence for the stability of the factor structure. Internal consistency was evaluated for both the overall scale and each subscale, demonstrating good to excellent reliability. Nevertheless, further studies using independent samples are required to confirm the stability of subscale reliability and the factor structure.

Third, the IPVNCS assesses self-perceived competencies, which may be influenced by social desirability or self-assessment bias. The scale does not measure actual clinical performance, and future studies should explore associations between IPVNCS scores and objective indicators of clinical practice.

Finally, the mandatory completion of all items, while preventing missing data, may have influenced response patterns. However, this approach ensured complete datasets for psychometric analysis.

## 6. Conclusions

The Intimate Partner Violence Competency Scale for Nurses (IPVNCS) is a theoretically grounded instrument with demonstrated initial construct validity and excellent internal consistency reliability. It is designed to accurately assess the skills of nurses in the detection, intervention, and support of victims of intimate partner violence. Aligned with the NIC 6403 nursing intervention (Table A1), the IPVNCS allows for a comprehensive and non-discriminatory assessment. The results of the validation confirm its reliability and usefulness for clinical practice, facilitating the identification of strengths and areas for improvement in the care of victims.

## 7. Future Research Directions

Future research should focus on the external validation of the IPVNCS in independent samples and diverse clinical and cultural settings to confirm its reliability and validity. Longitudinal studies are also needed to evaluate the sensitivity of the IPVNCS to changes over time, particularly in the context of targeted educational or training interventions.

Additionally, qualitative studies exploring nurses’ perceptions of the IPVNCS could provide valuable insights into its usability, acceptability, and integration into routine clinical practice. Comparative studies assessing the IPVNCS alongside existing IPV-related assessment tools may further clarify its relative strengths and potential applications.

Importantly, future implementation should emphasize the formative and supportive use of the IPVNCS, avoiding its perception as a punitive or evaluative tool. The scale is intended to support professional development, particularly for early-career nurses, and to inform institutional strategies aimed at improving IPV-related care. 

## Figures and Tables

**Table 1:** Descriptive characteristics of the sample.

Variable	n	%
Sex		
Male	34	16.8
Female	165	81.7
Age		
20–30	54	26.7
31–40	69	34.2
41–50	51	25.2
>50	28	13.9
Educational level		
Diploma	74	36.6
Bachelor’s degree	78	38.6
Postgraduate	44	21.8
Doctorate	6	3.0
Years of experience		
<5 years	49	24.3
6–10 years	30	14.9
11–15 years	48	23.8
>16 years	75	37.1
Primary occupation		
Primary care	75	37.1
Hospital care	99	49.0
Prehospital care	6	3.0
Teaching	8	4.0
Social care	11	5.4
School nurse	3	1.5
Total	202	100

**Table 2:** Summary item statistics.

	Mean	Minimum	Maximum	Rank	Maximum/Minimum	Variance	N
Element Averages	2.993	2.297	3.931	1.634	1.711	0.148	30
Element Variances	1.157	0.894	1.517	0.622	1.696	0.029	30
Covariances between elements	0.596	0.242	1.060	0.818	4.377	0.019	30
Correlations between elements	0.517	0.187	0.838	0.651	4.476	0.010	30

**Table 3:** Component Correlation Matrix. Extraction method: principal component analysis. Rotation method: Promax with Kaiser normalization.

Component	1	2	3	4
1	1.000	0.588	0.691	0.647
2	0.588	1.000	0.707	0.573
3	0.691	0.707	1.000	0.682
4	0.647	0.573	0.682	1.000

**Table 4:** Internal consistency (Cronbach’s alpha) of the IPVNCS total scale and subscales.

IPVNCS Dimension	Cronbach’s α
Total IPVNCS scale	0.970
Dimension 1: Intervention and Referral	0.905
Dimension 2: Detection and Assessment of Abuse	0.888
Dimension 3: Documentation and Record-keeping	0.919
Dimension 4: Psychosocial Support	0.900

**Table 5:** ANOVA with Friedman’s test and Tukey’s non-additivity test.

	Sum of Squares	df	Mean Square	Friedman’s Chi-Square	Sig.
Between subjects	3706.669	201	18.441		
Within subjects:					
Between elements	864.439 ^a^	29	29.808	1225.212	<0.001
Non-additive residue	20.038 ^b^	1	20.038	35.949	<0.001
Total	4133.067	5858	0.706		

^a^ Overall mean = 2.99. Concordance coefficient W=0.110. ^b^ Tukey’s estimate of the power at which observations must be made to obtain additivity = 1.583.

**Table 6:** KMO and Bartlett’s test.

Measure	Value
Kaiser–Meyer–Olkin (KMO)	0.947
Bartlett’s test ($\chi^{2}$)	5121.402
df	435
*p*-value	<0.001

**Table 7:** Confirmatory Factor Analysis (CFA).

	Absolute			Relative			Parsimony	
Model	$\chi^{2}$	$\chi^{2}$/df	RMSEA	NFI	TLI	CFI	AIC	RMR
Initial	0	3.055	0.101	0.77	0.82	0.83	1350.926	0.065
Final	0	2.066	0.073	0.86	0.91	0.92	720.736	0.52

## Data Availability

The original contributions presented in this study are included in the article. Further inquiries can be directed to the corresponding author.

## Supplementary Materials

The following supporting information can be downloaded at: https://www.mdpi.com/article/10.3390/jcm15031001/s1, Table S1: Intimate Partner Violence Nursing Competency Scale (IPVNCS) – English Version; Supplementary Material S1—Bilingual Questionnaire (Spanish—English); Informed Consent Statement Nursing Activities Assessment Scale (NIC 6403) on Intimate Partner Violence.

## Abbreviations

The following abbreviations are used in this manuscript:

IPV	Intimate partner violence
IPVNCS	Intimate Partner Violence Nursing Competency Scale

## Appendix A

**Table A1:** Nursing interventions against intimate partner violence, code. 6403: Support in the protection against partner abuse.

1. Investigate whether there are risk factors associated with domestic abuse (e.g., history of domestic violence, abuse, rejection, over-criticism, or feelings of worthlessness and lack of love; difficulty trusting others or feelings of lack of appreciation for others; feeling that asking for help is an indication of personal inadequacy; heightened need for physical care; many family caregiving responsibilities; substance use; depression; serious psychiatric illnesses; social isolation; poor relationship between partners; many marriages; pregnancy; poverty; unemployment; economic dependence; homelessness; infidelity; divorce, or death of a loved one). 2. Investigate whether there is a history of domestic abuse symptoms (e.g., numerous accidental injuries, multiple somatic symptoms, chronic abdominal pain, chronic headaches, pelvic pain, anxiety, depression, post-traumatic stress syndrome, and other psychiatric disorders). 3. Watch for signs and symptoms of physical abuse (e.g., numerous injuries at various stages of healing, unexplained lacerations, bruises or bruises, hairless areas on the head, tie marks on the wrists or ankles; “defensive” bruises on the forearms, and human bite marks). 4. Watch for signs and symptoms of sexual abuse (e.g., semen or dried blood, lesions on the external genitalia, sexually transmitted diseases, or dramatic behavior or health changes of undetermined etiology). 5. Watch for signs and symptoms of emotional abuse (e.g., low self-esteem, depression, humiliation, and feelings of defeat; overly cautious behavior toward a partner; self-harm or suicidal attitudes). 6. Watch for signs and symptoms of exploitation (e.g., inadequate provision of basic needs when adequate resources are available, deprivation of personal possessions, unexplained loss of social assistance, lack of knowledge of personal finances or legal matters). 7. Document evidence of physical abuse using standardized assessment tools and photographs. 8. Document evidence of sexual abuse using standardized assessment tools and photographs. 9. Listen carefully to the person who begins to talk about his or her own problems. 10. Identify inconsistencies in the explanation of the cause of the injuries. 11. Determine the correlation between the type of injury and the description of the cause. 12. Interview the patient and/or someone else who knows the situation about the alleged abuse in the absence of the partner. 13. Encourage the admission of the patient for better observation and study, when appropriate. 14. Observe and record partner interactions, as appropriate (e.g., recording the hours and duration of partner visits during hospitalization, insufficient or exaggerated reactions by the partner). 15. Observe if the individual shows excessive submission, such as passively submitting to hospital procedures. 16. Observe if there is a progressive deterioration of the physical and/or emotional state of individuals. 17. Observe if visits to clinics, emergencies or consultations with the doctor for minor problems are repeated. 18. Establish a system for marking individual medical records in which there are suspicions of abuse. 19. Provide positive affirmations about worth. 20. Encourage the expression of concerns and feelings, including fear, guilt, shame, and self-blame. 21. Provide support for victims to take action and make changes to prevent further retaliation. 22. Help individuals and their families develop coping strategies in stressful situations. 23. Help individuals and their families objectively assess the strengths and weaknesses of relationships. 24. Refer individuals at risk of abuse or those who have suffered abuse to appropriate specialists and services (e.g., public health services, social services, counseling, legal assistance). 25. Refer the abusing spouse to appropriate specialists and services. 26. Provide confidential information regarding shelters for people experiencing domestic violence, as appropriate. 27. Initiate the development of a security plan to use if violence escalates. 28. Report any situation where abuse is suspected in accordance with mandatory reporting laws. 29. Initiate community education programs designed to reduce violence. 30. Observe the use of resources of the community
