# Supplementary material for: Development and Validation of the Intimate Partner Violence Nursing Competency Scale (IPVNCS): A Psychometric Tool to Strengthen Clinical Detection and Intervention
Source: J Clin Med. 2026 Jan 26;15(3):1001. doi: 10.3390/jcm15031001 (PMC12898863; doi:10.3390/jcm15031001)
Supplement: Supplementary file 1 [file jcm-15-01001-s001.zip › jcm-4074177-supplementary/Informed_Consent_English.pdf]

Informed Consent Statement
Nursing Activities Assessment Scale (NIC 6403) on Intimate Partner Violence

Study Description

This assessment forms part of a doctoral thesis in Nursing on intimate partner violence at CEU San Pablo University.

The questionnaire is anonymous and can be completed in less than five minutes.

The scale evaluates high-risk domestic dependency relationships and nursing actions aimed at preventing further physical, sexual, emotional harm or exploitation within intimate partner relationships. The purpose of this questionnaire is to assess nurses' ability to detect intimate partner violence (IPV) in individuals of any sex.

Principal Investigator: david.caserobenavente@ceu.es

Instructions

Please indicate, for each item, your level of personal ability or difficulty in performing the listed nursing activities in your daily clinical practice.

Informed Consent

I hereby declare that I am of legal age and have full capacity to provide consent. I voluntarily and expressly agree to participate in this anonymous online health-related questionnaire.

I have been informed that the purpose of this scale is to evaluate nursing practice in situations of intimate partner and domestic violence, and that the results will be used exclusively for academic research and for the development and publication of a doctoral thesis, without any commercial purpose.

I understand and acknowledge that:

• The questionnaire is entirely anonymous; no personal identifying data will be recorded or used at any time.

• Participation is voluntary, and I may withdraw my consent at any moment without any consequences for my rights, well-being, or professional activity.

• This questionnaire involves no risks to my physical or mental health.

Accordingly, I freely, knowingly and explicitly provide my consent to participate in this anonymous online health-related questionnaire. This informed consent complies with the principles of the EU General Data Protection Regulation (GDPR).
