# Supplementary material for: Development and Validation of the Intimate Partner Violence Nursing Competency Scale (IPVNCS): A Psychometric Tool to Strengthen Clinical Detection and Intervention
Source: J Clin Med. 2026 Jan 26;15(3):1001. doi: 10.3390/jcm15031001 (PMC12898863; doi:10.3390/jcm15031001)
Supplement: Supplementary file 1 [file jcm-15-01001-s001.zip › jcm-4074177-supplementary/Supplementary Table S1.pdf]

Supplementary Table S1. Intimate Partner Violence Nursing Competency Scale (IPVNCS) – English Version

Instructions:

Please indicate your perceived level of competence for each of the following nursing activities related to the clinical management of intimate partner violence (IPV).

Response scale:

1 = Not competent at all

2 = Slightly competent

3 = Moderately competent

4 = Very competent

5 = Highly competent

Dimension 1: Detection and Assessment

1. I am able to identify risk factors associated with intimate partner violence.
2. I am able to recognize physical signs and symptoms suggestive of intimate partner violence.
3. I am able to recognize psychological and emotional indicators associated with intimate partner violence.
4. I am able to detect signs of sexual abuse related to intimate partner violence.
5. I am able to identify inconsistencies between reported injuries and clinical findings.
6. I am able to assess the progression or worsening of physical or emotional conditions potentially related to intimate partner violence.

Dimension 2: Documentation and Recording

7. I am competent in documenting suspected cases of intimate partner violence using standardized clinical records.
8. I am able to accurately record physical evidence related to intimate partner violence.
9. I am able to document psychosocial findings relevant to intimate partner violence.

10. I am able to maintain clear, objective, and legally appropriate documentation in cases of intimate partner violence.
11. I am able to record observations of patient–partner interactions when intimate partner violence is suspected.
12. I am able to ensure confidentiality and data protection when documenting intimate partner violence cases.

Dimension 3: Psychosocial Support

13. I am able to listen actively and empathetically to patients who disclose intimate partner violence.
14. I am able to provide emotional support to patients experiencing intimate partner violence.
15. I am able to encourage patients to express feelings such as fear, guilt, or shame related to intimate partner violence.
16. I am able to help patients identify personal strengths and coping strategies in situations of intimate partner violence.
17. I am able to support patients in making decisions aimed at preventing further violence.
18. I am able to assess psychosocial needs associated with intimate partner violence.

Dimension 4: Intervention and Referral

19. I am able to initiate appropriate referrals to specialized healthcare, social, or legal services for patients experiencing intimate partner violence.
20. I am able to provide patients with information about available community resources related to intimate partner violence.
21. I am able to collaborate with multidisciplinary teams in the management of intimate partner violence cases.
22. I am able to contribute to the development of safety plans for patients at risk of intimate partner violence.
23. I am able to follow institutional protocols for reporting suspected intimate partner violence.
24. I am able to act in accordance with legal and ethical requirements when managing intimate partner violence cases.
25. I am able to support patients during referral processes to external services.
26. I am able to participate in preventive or educational initiatives related to intimate partner violence.

Scoring

The IPVNCS total score is calculated by summing the scores of all items, with higher scores indicating higher perceived nursing competence in the clinical management of intimate partner violence. Subscale scores can be calculated by summing items corresponding to each dimension.
