# Supplementary material for: Development and Validation of the Intimate Partner Violence Nursing Competency Scale (IPVNCS): A Psychometric Tool to Strengthen Clinical Detection and Intervention
Source: J Clin Med. 2026 Jan 26;15(3):1001. doi: 10.3390/jcm15031001 (PMC12898863; doi:10.3390/jcm15031001)
Supplement: Supplementary file 1 [file jcm-15-01001-s001.zip › jcm-4074177-supplementary/Supplementary_S1_Bilingual_Table.pdf]

Supplementary Material S1 – Bilingual Questionnaire (Spanish–English)

Spanish	English
Observar signos y síntomas de abuso físico.	Observe signs and symptoms of physical abuse.
Observar signos y síntomas de abuso sexual.	Observe signs and symptoms of sexual abuse.
Observar signos y síntomas de explotación.	Observe signs and symptoms of exploitation.
Observar signos y síntomas de abuso emocional.	Observe signs and symptoms of emotional abuse.
Observar sumisión excesiva.	Observe excessive submissiveness.
Observar e interpretar interacciones de pareja.	Observe and record partner interactions.
Observar deterioro físico progresivo.	Observe progressive physical deterioration.
Observar deterioro emocional progresivo.	Observe progressive emotional deterioration.
Observar visitas repetidas por problemas menores.	Observe repeated visits for minor issues.
Observar el uso de recursos comunitarios.	Observe the use of community resources.
Documentar evidencia de abuso físico o sexual.	Document evidence of physical or sexual abuse.
Escuchar activamente cuando la persona expresa preocupaciones.	Listen attentively when the person expresses concerns.
Investigar factores de riesgo asociados al abuso doméstico.	Investigate risk factors associated with domestic abuse.
Investigar historial de síntomas relacionados con abuso.	Investigate history of symptoms related to domestic abuse.
Identificar inconsistencias en la explicación de lesiones.	Identify inconsistencies in the injury explanation.
Determinar correlación entre tipo de lesión y causa reportada.	Determine correlation between injury type and cause.
Entrevistar sin la presencia de la pareja.	Interview without the partner present.
Fomentar ingreso hospitalario cuando sea necesario.	Encourage hospital admission when appropriate.
Establecer alertas en historias clínicas.	Flag medical records with suspected abuse.
Favorecer la expresión de emociones y preocupaciones.	Encourage expression of emotions and concerns.
Proporcionar afirmaciones positivas.	Provide positive affirmations.
Apoyar a víctimas en acciones protectoras.	Support victims in protective action.
Ayudar a desarrollar estrategias de afrontamiento.	Help develop coping strategies.
Ayudar a evaluar relaciones interpersonales.	Help evaluate relationship strengths and weaknesses.
Derivar a víctimas a servicios especializados.	Refer victims to specialized services.
Derivar a la pareja agresora a servicios correspondientes.	Refer the abusive partner to appropriate services.
Informar sobre recursos y alojamientos seguros.	Provide information about shelters and safe housing.
Desarrollar un plan de seguridad.	Develop a safety plan.

Impulsar programas educativos comunitarios.	Initiate community education programs.
Observar el uso de recursos disponibles.	Observe the use of available resources.
